# MetaDiff: differential isoform expression analysis using random-effects meta-regression

**DOI:** 10.1186/s12859-015-0623-z

**Published:** 2015-07-02

**Authors:** Cheng Jia, Weihua Guan, Amy Yang, Rui Xiao, W. H. Wilson Tang, Christine S. Moravec, Kenneth B. Margulies, Thomas P. Cappola, Chun Li, Mingyao Li

**Affiliations:** 1 Department of Biostatistics and Epidemiology, University of Pennsylvania Perelman School of Medicine, Philadelphia, PA 19104 USA; 2 Division of Biostatistics, School of Public Health, University of Minnesota School of Public Health, Minneapolis, MN 55455 USA; 3 Department of Cardiovascular Medicine, Cleveland Clinic, Cleveland, OH 44195 USA; 4 Cardiovascular Institute, University of Pennsylvania Perelman School of Medicine, Philadelphia, PA 19104 USA; 5 Department of Epidemiology and Biostatistics, Case Western Reserve University, Cleveland, OH 44106 USA

**Keywords:** RNA-Seq, Isoform, Differential expression, Random-effects meta-regression

## Abstract

**Background:**

RNA sequencing (RNA-Seq) allows an unbiased survey of the entire transcriptome in a high-throughput manner. A major application of RNA-Seq is to detect differential isoform expression across experimental conditions, which is of great biological interest due to its direct relevance to protein function and disease pathogenesis. Detection of differential isoform expression is challenging because of uncertainty in isoform expression estimation owing to ambiguous reads and variability in precision of the estimates across samples. It is desirable to have a method that can account for these issues and is flexible enough to allow adjustment for covariates.

**Results:**

In this paper, we present MetaDiff, a random-effects meta-regression model that naturally fits for the above purposes. Through extensive simulations and analysis of an RNA-Seq dataset on human heart failure, we show that the random-effects meta-regression approach is computationally fast, reliable, and can improve the power of differential expression analysis while controlling for false positives due to the effect of covariates or confounding variables. In contrast, several existing methods either fail to control false discovery rate or have reduced power in the presence of covariates or confounding variables. The source code, compiled JAR package and documentation of MetaDiff are freely available at https://github.com/jiach/MetaDiff.

**Conclusion:**

Our results indicate that random-effects meta-regression offers a flexible framework for differential expression analysis of isoforms, particularly when gene expression is influenced by other variables.

**Electronic supplementary material:**

The online version of this article (doi:10.1186/s12859-015-0623-z) contains supplementary material, which is available to authorized users.

## Background

The advent of massively parallel sequencing has revolutionized genetics, epigenetics, and transcriptomics studies. RNA sequencing (RNA-Seq) allows an unbiased survey of the entire transcriptome in a high-throughput manner. It has rapidly replaced traditional microarrays as the major platform for transcriptomics studies because it allows for more accurate and a wider range of measurement of gene expression [1].

A major application of RNA-Seq is to detect differential isoform (i.e., transcript) expression across experimental conditions, which is of great biological interest due to its direct relevance to protein function and disease pathogenesis. Recent evidence suggests that almost all human multi-exon genes have more than one isoform [2], and different isoforms are often differentially expressed (DE) across tissues, developmental stages, disease conditions, and even across cells from the same tissue [3, 4]. Therefore, detection of DE isoforms is important for understanding complicated biological mechanisms and for mapping disease susceptibility genes.

Detection of DE isoforms using RNA-Seq, however, is challenging because of the uncertainty in isoform expression estimation owing to ambiguous reads and the variability in precision of the estimates across samples. Popular analysis methods such as DESeq [5], DESeq2 [6], and EdgeR [7] expect read counts as input. When using these programs, one would have to estimate the number of fragments originating from each isoform using other programs [8–10] and then analyze the estimated counts as if they were directly observed. Since the estimation of isoform expression may be biased systematically, failing to account for the variability in estimation precision can result in increased false discoveries. Other programs such as Cuffdiff [11], BitSeq [12], and EBSeq [13] can appropriately account for uncertainty in isoform expression estimates. However, these programs cannot be used to adjust for covariates and confounders that may affect gene expression. Recent studies have shown that gene expression can change with environment and age in multiple tissues [14, 15]; for example, Glass et al. [15] found that ~10-15 % of the genes in the human genome are affected by age in skin, adipose, blood, and brain. In this paper, we refer a covariate as an independent variable that correlates with gene expression but not with the phenotype of interest (e.g., disease status, or treatment group), while a confounder as one that correlates with both gene expression and the phenotype.

A method for detecting DE isoforms ideally should be able to: 1) account for isoform expression estimation uncertainty, 2) account for variation in the precision of isoform expression estimates across biological replicates, and 3) allow adjustment for covariates and confounding factors. Recently, Turro et al. [16] developed MMDIFF, a Bayesian random-effects method that meets these purposes. However, it is difficult to directly compare MMDIFF with other alternative methods that are frequentist in nature and allow assessment of statistical significance through p-values.

**Fig. 1:**
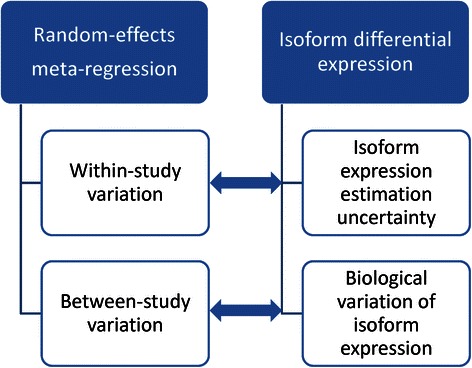
Analogy between meta-regression and isoform differential expression analysis in RNA-Seq

In this paper, we present MetaDiff, a regression framework based on a random-effects meta-regression model that can be considered as a frequentist version of MMDIFF [16]. The original goal of random-effects meta-regression is to synthesize results from multiple studies while accounting for varying standard errors of the effect estimates by explicitly allowing for different sources of variability: within- and between-study variation. Its mathematical model matches perfectly with the analysis of DE isoform in that within-study variation represents variable precision in isoform expression estimation and between-study variation represents variation in isoform expression levels across samples (Fig. 1). This analogy motivated us to explore random-effects meta-regression as a means for identification of DE isoforms.

Through extensive simulations and the analysis of an RNA-Seq dataset on human heart failure, we show that MetaDiff is computationally fast, reliable, and can easily incorporate covariates/confounders. In summary, random-effects meta-regression offers a flexible framework for differential expression analysis of isoforms, particularly when gene expression is influenced by other variables.

## Results

We evaluated the performance of random-effects meta-regression on both simulated and real RNA-Seq data and compared it with five existing algorithms, including Cuffdiff, DESeq, DESeq2, EdgeR and EBSeq. To make a fair comparison, all programs used FPKMs estimated from Cufflinks, one of the most popular programs for isoform expression estimation. Since DESeq, DESeq2, EdgeR and EBSeq can only take counts as input, we converted the estimated FPKMs into counts for these programs. EBSeq does not explicitly model estimation uncertainty for each isoform, but rather divides isoforms into different groups according to the number of isoforms in a gene. In our analysis, we used the default option in EBSeq that divides the isoforms into three groups. A transcript was declared to be DE if its FDR-adjusted p-value was less than the nominal FDR level (for Cufflinks, DESeq, DESeq2, and EdgeR) or if its posterior probability of DE was greater than one – nominal FDR level (for EBSeq). We did not include MMDIFF in the comparison because it can only take isoform expression estimates obtained from MMSEQ [17]. We note that DESeq, DESeq2, and EdgeR are not designed for differential expression analysis of isoforms. However, given the popularity of these methods, it might be tempting for an end user to identify DE isoforms using FPKM converted counts. Therefore, we included DESeq, DESeq2, and EdgeR in our comparisons.

### Overview of random-effects meta-regression

Isoform-specific gene expression levels cannot be directly observed in RNA-Seq, but rather have to be estimated using deconvolution-based algorithms [8–10]. A natural way to account for variable uncertainty in isoform expression estimation when testing the relationship between isoform expression level *Y*_*i*_ and the variable of interest *X*_*i*_ (e.g., disease status, treatment group, etc.) is to use a random-effects regression model, which can be written as

$$ \log \left({Y}_i\right)={\beta}_0+{\beta}_1{X}_i+{\beta}_2{Z}_i+{u}_i+{e}_i. $$

Here *i* is an index for subject (*i* = 1, …, *n*), *u*_*i*_ represents the estimation uncertainty for log(*Y*_*i*_), and *e*_*i*_ is random error. To test the null hypothesis of no differential expression between groups, i.e., H_0_ : *β*_1_ = 0, we use two statistical tests: *t*-test and Bartlett corrected likelihood-ratio test (BcLR) [18]. Details of the random-effects meta-regression model are described in Methods.

### RNA-Seq data simulation

We conducted simulations to evaluate the performance of random-effects meta-regression and compared it with other state-of-the-art algorithms for differential expression analysis. To simulate a realistic dataset, we used Flux Simulator [19] to generate paired-end RNA-Seq data by modeling RNA-Seq experiments *in silico*. The human genome sequence (hg19, NCBI build 37) was downloaded from UCSC together with the coordinates of all isoforms in the RefGene table. Flux Simulator assigns an abundance value to each isoform following a mixed power/exponential law. Additionally, it simulates common sources of systematic bias in the abundance and distribution of reads by *in silico* library preparation and sequencing. We simulated 16 cases and 16 controls. To reflect varying sequencing depth across samples, each individual’s total sequencing output was determined by a uniform distribution from 8 million to 12 million reads.

We first simulated the data without any covariate or confounding effect (Scenario I). We designated 30 % of the transcripts to be DE, half up-regulated in cases by 1.25 fold and half down-regulated by 1.25 fold. The remaining 70 % of the transcripts were assumed to be non-DE between the two groups. This setup allowed us to evaluate both false discovery rate (FDR) and power.

**Fig. 2:**
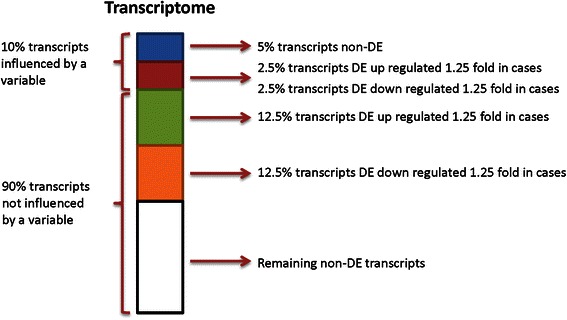
Simulation setup. Simulation setup when isoform expression is influenced by a variable either as a covariate (Scenario II) or as a confounder (Scenario III)

Next, we considered a situation in which some gene expression levels were influenced by a covariate (e.g., age) (Scenario II). The distribution of the covariate was uniform from 18 to 60 in both cases and controls. Similar to Scenario I, 30 % of the transcripts were designated to be DE and 70 % were non-DE. We further allowed 10 % of the transcripts to be affected by the covariate, half with differential expression between the cases and controls and half without differential expression; these transcripts had 1.35 fold increased expression for every one standard deviation increase in the covariate, which is equivalent to 2.5 % increased expression for every one unit increase of the covariate. Detailed simulation setup is shown in Fig. 2.

Finally, we considered a variation of Scenario II, in which the covariate was correlated with both gene expression and disease status (Scenario III). We introduced confounding by allowing the covariate to have different distributions: uniform(40,85) for the cases and uniform(18,60) for the controls. The rest of the simulation setup was the same as that in Scenario II (Fig. 2).

For each simulated dataset, the RNA-Seq reads were mapped to the human reference genome using Tophat with default options [20]. Isoform-specific gene expression was estimated using Cufflinks [8]. The estimated FPKM was normalized following the procedure described in DESeq [5]. For each expressed transcript, we tested for differential expression between cases and controls using the *t*-test and the BcLR test implemented in the R *metatest* package. We also analyzed the data using popular software packages including Cuffdiff (version 2.1.1), DESeq (version 1.20.0), DESeq2 (version 1.16), EdgeR (version 3.10.0), and EBSeq. Since DESeq, DESeq2, EdgeR and EBSeq can only take counts as input, we converted isoform expression to read count using the formula *FPKM* × *N* × *L*/10^9^, where *FPKM* is the estimated expression level, *N* is the total number of mapped reads in the sample, and *L* is the length of the transcript. We note that Cuffdiff and EBSeq cannot adjust for covariates. For each of the three scenarios, we also evaluated the impact of sample size by analyzing a subset of *m* cases and *m* controls (*m* = 4, 8) randomly chosen from the simulated dataset. We note that Cuffdiff uses a sampling-based method for differential analysis and only generates p-values ≥ 5 × 10^−5^.

### Comparison of empirical FDR in simulated data

**Fig. 3:**
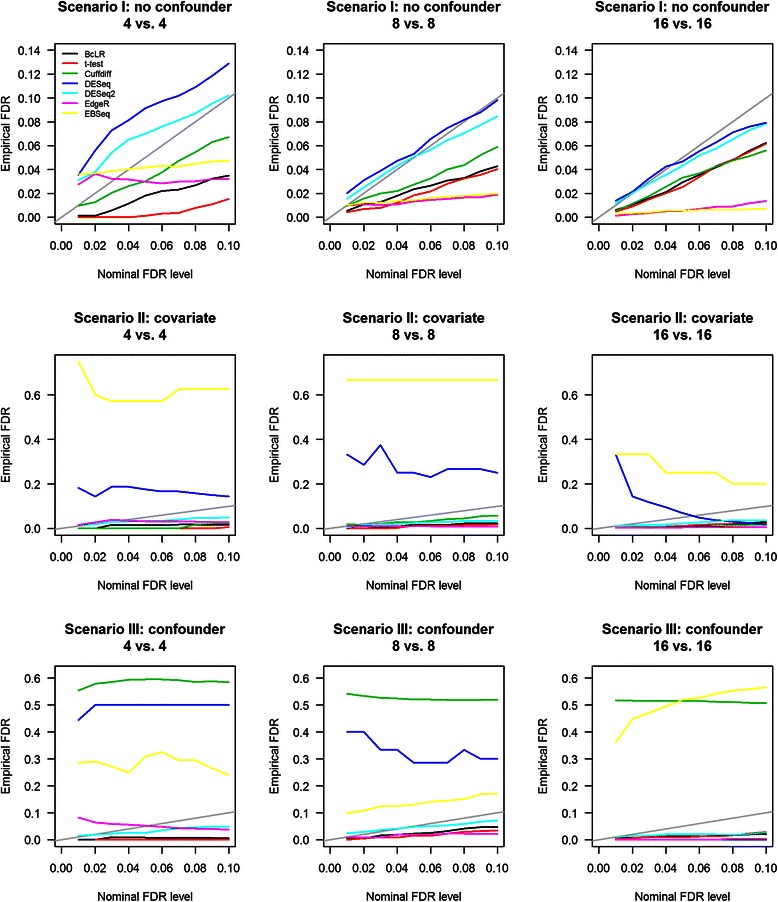
Empirical FDR of different tests in detecting DE isoforms at various nominal FDR levels. Empirical FDR for Scenario I was calculated using all isoforms, whereas the empirical FDR for Scenarios II and III was calculated using only those isoforms that were influenced by a covariate or a confounder

The empirical FDR was estimated as the fraction of true non-DE transcripts among those declared to be DE. For meta-regression based tests, we filtered out transcripts for which the normalized FPKM had coefficient of variation (CV) >0.4 in either cases or controls as these transcripts would likely yield false positive findings due to high heteroscedasticity (Additional file 1: Figures S1-S7). Fig. 3 shows the empirical FDR of each method across a range of nominal FDR levels (0.01 to 0.1 with increment of 0.01). When there was no covariate or confounding variable (Scenario I), all methods had well-controlled FDR except for DESeq and DESeq2 in which the empirical FDRs were well-above the nominal levels when sample size was small (*m* = 4). This is consistent with findings reported in the DESeq2 paper, which reported that DESeq and DESeq2 tend to have inflated FDRs when sample size is small. When there was a covariate that influenced gene expression (Scenario II), the empirical FDRs of EBSeq and DESeq were severely inflated, regardless of the sample size, whereas all other methods had empirical FDRs below the nominal levels. In scenario III, when there was a confounder variable, Cuffdiff, EBSeq, and DESeq yielded highly inflated empirical FDRs due to their inability to adjust for confounders. DESeq’s empirical FDR became smaller when sample size was increased to 16. In contrast, BcLR, *t*-test, DESeq2, and EdgeR all had empirical FDRs well below or close to the nominal levels. These results clearly demonstrate the importance of adjusting for confounding variables in differential expression analysis.

### Comparison of quantile-quantile(QQ) plots in simulated data

**Fig. 4:**
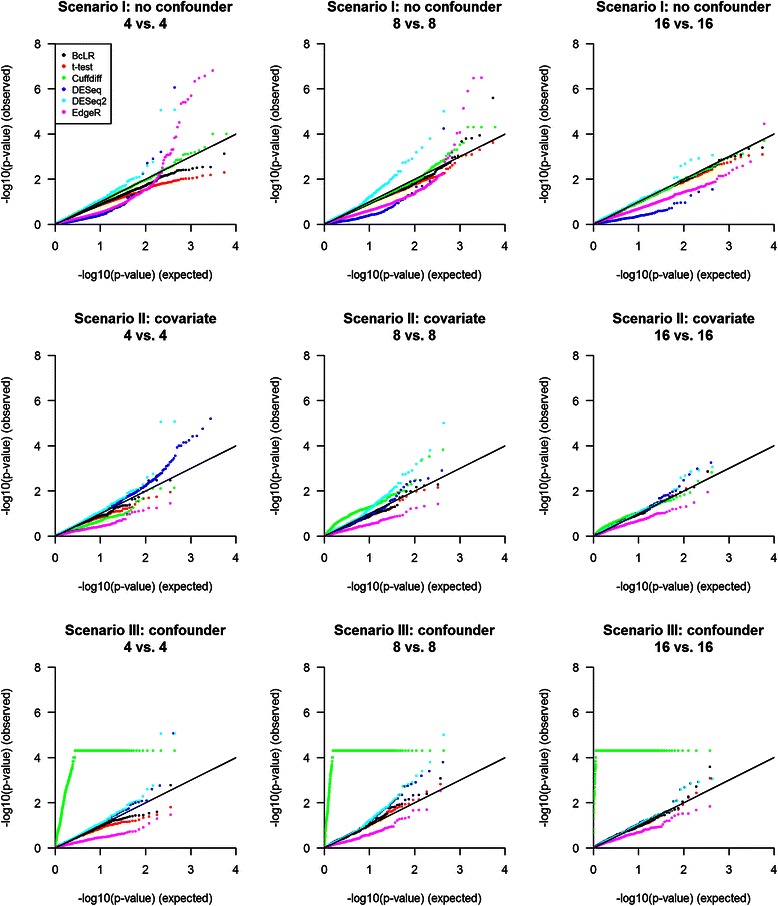
Quantile-quantile (QQ) plots of different tests in detecting DE isoforms. Displayed are p-values for those true non-DE isoforms

We also examined the QQ plots for non-DE transcripts (Fig. 4). A good-performing method is expected to have –log10 transformed p-values falling along the diagonal line in a QQ plot. In general, BcLR and *t*-test had p-values close to the expected distribution in all three scenarios. However, some of the other methods had strong deviation from the diagonal line even though their empirical FDRs were under control. EdegR showed upward deviation in Scenario I when sample size was small (*m* = 4) or moderate (*m* = 8). DESeq and DESeq2 tended to deviate upward in all three scenarios, and the degree of deviation was more pronounced in Scenario I. Such deviation for EdgeR, DESeq, and DESeq2 is likely due to their inability to account for isoform expression estimation uncertainty. In Scenario III, Cuffdiff showed a substantial upward deviation from the diagonal line, which is consistent with its highly inflated FDRs shown in Fig. 3. The plateau is due to the sampling based method employed by Cuffdiff for significance evaluation; the current program only gives p-values ≥ 5 × 10^−5^.

### Comparison of power in simulated data

**Fig. 5:**
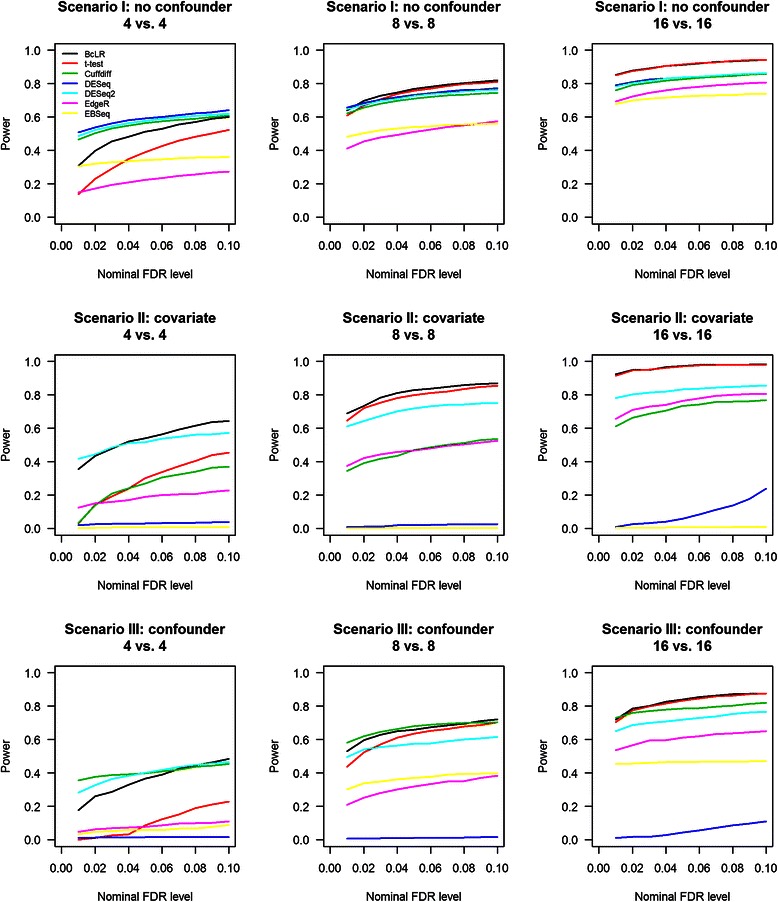
Estimated power of different tests in detecting DE isoforms at various nominal FDR. Power for Scenario I was calculated using all true DE isoforms, whereas the power for Scenarios II and III was calculated using only those true DE isoforms that were influenced by a covariate or a confounder

**Fig. 6:**
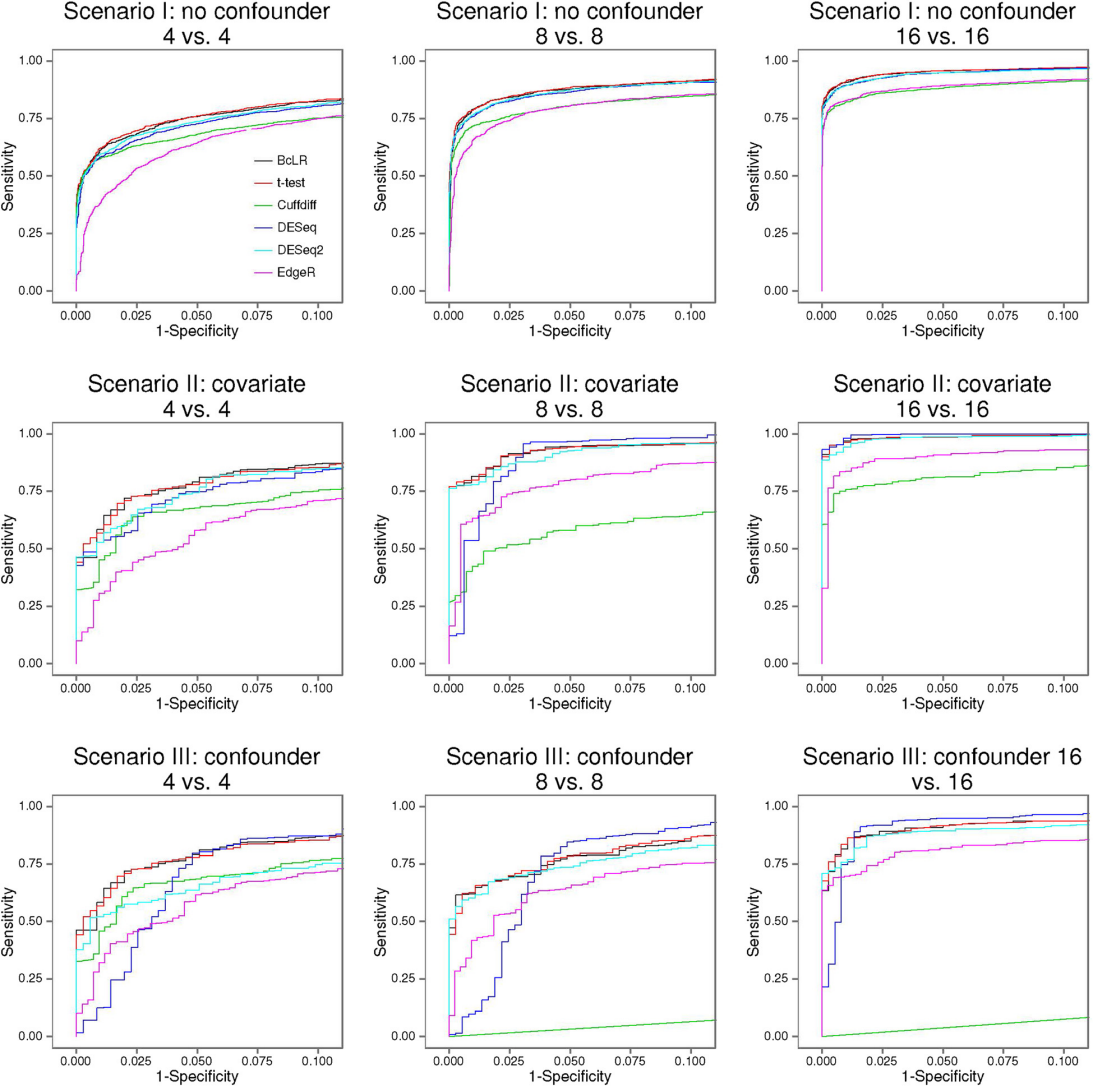
Receiver Operating Characteristic (ROC) curves. Sensitivity and specificity were calculated by varying the p-value cutoffs

Next, we compared the power of different methods in detecting DE isoforms across a range of nominal FDR levels (Fig. 5). In Scenario I, BcLR and *t*-test had the highest power when sample size was eight or 16, whereas EdgeR and EBSeq had relatively lower power than the other methods. When sample size was four, DESeq, DESeq2, and Cuffdiff had the highest power, but this should be interpreted with caution because DESeq and DESeq2’s empirical FDRs exceeded the nominal levels. When sample size was four, Cuffdiff performed the best in that its power was the third highest, its empirical FDR was under control and its p-value distribution agreed well with the expected null distribution in the QQ plot. In Scenario II, BcLR and *t*-test had the highest power when sample size was eight or 16, followed by DESeq2, EdgeR, and Cuffdiff, whereas DESeq and EBSeq had almost no power. When sample size was four, BcLR had the highest power, followed by DESeq2, but it is worth noting that DESeq2 tended to generate extremely small p-values when sample size was small, thus one should interpret the results from DESeq2 with caution. Since DESeq and EBSeq had almost no power in detecting in DE isoforms in Scenario II, as a sanity check, we examined those isoforms that were not influenced by the covariate. As expected, DESeq and EBSeq had similar power as those shown in Scenario I. These results indicate that DESeq and EBSeq may not be robust when the expression of a gene is influenced by covariates. In Scenario III, BcLR appeared to be the best-performing method when sample size was eight or 16, followed by *t*-test, DESeq2, EdgeR, and EBSeq, and DESeq still had almost no power. When sample size was four, DESeq2 had slightly higher power than BcLR, but that is likely due to its relative higher FDR and upward deviation from the diagonal line in the QQ plot. We further compared the performance of different methods by using Receiver Operating Characteristic (ROC) curve (Fig. 6; Additional file 1: Figure S8). The relative performance of different methods is generally consistent with our power comparison results.

### Application to the human heart failure RNA-Seq data

We next evaluated the performance of these methods using a dataset from our ongoing study on human heart failure. RNA sequencing using Illumina HiSeq 2000 was performed on left ventricle samples collected from four cases and three controls in the MAGNet study (http://www.med.upenn.edu/magnet). The heart was perfused with cold cardioplegia prior to cardiectomy to arrest contraction and prevent ischemic damage. Left ventricular free-wall tissue was harvested and snap frozen with liquid nitrogen at the time of cardiac surgery from subjects with heart failure undergoing transplantation and from unused donor hearts. This study was approved by the University of Pennsylvania Institutional Review Board and the Cleveland Clinic Institutional Review Board. All participants were 18 years or older and provided written informed consent. The seven selected subjects had a wide range of age distribution (controls: mean = 47, range [32, 57]; cases: mean = 58.5, range [41, 68]). Among the seven subjects, four were male and three were female. Poly-A library preparation and sequencing was performed at the Penn Genome Frontiers Institute’s High-Throughput Sequencing Facility following standard protocols. The average sequencing depth was 43 million 2 × 101 bp paired-end reads. We mapped the reads to the human reference genome using Tophat, and then applied several filtering criteria to ensure that the reads were uniquely mapped with a mapping quality score ≥ 30 and that two reads in a pair were mapped to the same chromosome with expected orientations and distance < 500,000 bp. We estimated isoform-specific gene expression using Cufflinks.

In this heart failure dataset, the controls tended to be younger than the cases. Since men have a higher incidence of heart failure than women [21], both sex and age may influence gene expression in heart. We performed two sets of analyses, both at isoform level, one without any covariate adjustment and one with adjustment for age and sex. The numbers of DE transcripts detected by each test are shown in Table 1. Among the 94 transcripts detected by age-sex adjusted BcLR test but missed by unadjusted BcLR test, most (71/94) had p-values less than 0.05 for age or sex, suggesting the influence of covariates/confounders on isoform expression in heart. To assess the impact of covariate adjustment, we also compared the p-values between unadjusted and adjusted analyses. The Spearman correlation between unadjusted p-values and age-sex adjusted p-values was 0.66 for BcLR, 0.67 for *t*-test, 0.85 for DESeq, 0.64 for DESeq2 and 0.65 for EdgeR. If age and sex influence gene expression in heart, the correlation may not be very high.

**Table 1:** Number of DE transcripts (FDR adjusted p-value < 0.05 or posterior probability of DE > 0.95) detected by each method in the heart failure dataset

	Unadjusted	Age-sex-adjusted	Overlap
BcLR	6	95	1
*t*-test	1	0	0
DESeq	106	77	56
DeSeq2	102	49	31
EdgeR	3	0	0
Cuffdiff	7	-	-
EBSeq	256	-	-

We further examined the 41 DE transcripts that were detected by the age-sex adjusted BcLR test but were missed by the other tests, including age-sex adjusted *t*-test, DESeq, DESeq2 and EdgeR, and unadjusted BcLR, *t*-test, DESeq, DESeq2 and EdgeR. Several of these transcripts are from genes that have been implicated in heart failure pathogenesis. For example, Schattermann et al. [22] reported that *PDGFA* is required for normal murine cardiovascular development. Wang et al. [23] showed that *PLCE1* is upregulated in human hearts during heart failure, which is consistent with elevated expression levels of the DE isoform (NM_001165979) observed in our heart failure samples. Proteins encoded by the *SMYD* family are shown to be key regulators in skeletal and cardiac muscle development and function [24]. *TMEM88* encodes a protein that is crucial for heart development and acts downstream of GATA factors in the pre-cardiac mesoderm. It is worth noting that among the genes for which the 41 DE transcripts originate from, only about half were detected to be DE by gene level analysis using the age-sex adjusted BcLR test. This suggests that if one were to perform gene-only analysis, signals at the isoform level would have been missed.

## Discussion

One of the major applications of RNA-Seq is to identify DE isoforms. In differential expression analysis, it is important to account for the fact that isoform expression levels are estimated rather than observed, that they are estimated with various precision across samples, and that covariates and confounding factors may play a role to influence gene expression. To do so, we have proposed a flexible regression framework, utilizing the well-established random-effects meta-regression approach. Through computer simulations and the analysis of a real RNA-Seq dataset on human heart failure, we demonstrated that the proposed method can improve the power of isoform differential analysis while controlling for false positives due to the effect of covariates or confounding variables. The meta-regression approach we used is computationally efficient and widely available in existing statistical software packages. We have provided a tool and instructions on how to use meta-regression for isoform differential expression analysis with RNA-Seq data.

We have compared the performance of our method and other commonly used methods for differential expression analysis, including Cuffdiff, DESeq, DESeq2, EdgeR, and EBSeq. Both Cuffdiff and EBSeq take into account the estimation uncertainty for isoform expression levels, although EBSeq does not explicitly model the degree of uncertainty. In our simulated data, when no covariate and confounder influenced isoform expression (Scenario I), Cuffdiff had lower power than our method when *m* = 8 or 16, but better power when sample size was small (*m* = 4). In contrast, EBSeq had conservative FDR among non-DE transcripts and correspondingly lower power for detecting true DE transcripts. When a covariate or confounder was present, these two methods showed either lower statistical power (in Scenario II) or inflated FDR (in Scenario III) as they are unable to adjust for covariates.

DESeq, DESeq2, and EdgeR are based on negative binomial regression to model read counts from an RNA-Seq experiment, and thus it is natural for these methods to incorporate covariates. To estimate the variance in a negative binomial distribution, EdgeR relates the variance to the mean through a dispersion parameter that is constant across all genes. DESeq, on the other hand, applies a local regression to allow the dispersion parameter to vary from gene to gene. DESeq2 is a successor of DESeq. It uses shrinkage estimation to estimate dispersion parameters and fold change, thus offering improved stability as compared to DESeq. Additionally, DESeq2 allows adjustment of continuous covariates, whereas DESeq can only adjust discrete covariates. In our simulations, although EdgeR could control FDR in the presence of a confounder, it was conservative in all three scenarios. It also had lower power to detect true DE transcripts compared to our method and Cuffdiff in Scenario I. In contrast, DESeq showed inflated FDR for non-DE transcripts regardless the presence of confounder, especially when sample size was small. When a covariate or a confounder was present, DESeq had little power to detect DE transcripts that were correlated with the covariate. DESeq2 showed better performance than DESeq, however, its overall performance was not as satisfactory as BcLR and *t*-test. We note that EdgeR, DESeq, and DESeq2 cannot take into account the uncertainty in isoform expression estimation, which may lead to biased testing results.

The uncertainty in an isoform FPKM estimate can be quantified as a standard error, which can be calculated in Cufflinks. In differential expression analysis, the FPKM value is usually log-transformed, and we approximate the variance of log(FPKM) using the delta method, which is also used in Cuffdiff 1.0 [8]. However, this approximation can be poor and lead to false positive results when the variance of FPKM value is large compared to its magnitude. Hence we filtered out transcripts with large CVs in meta-regression. To avoid using delta method to approximate the variance of log(FPKM), one could use MMSEQ estimated isoform expression because MMSEQ directly gives the variance estimate of log(FPKM). Since DE transcripts that were influenced by a covariate or a confounder tended to have a higher CV than those not influenced by covariates/confounders, the true DE transcripts that were filtered out would have a higher proportion in the former. This is confirmed in Additional file 2: Table S1. If we had not applied the filter, the power of our proposed approach would have been even higher, but at the expense of inflated false positive results. We are in search of alternative filtering criteria to minimize the number of true DE transcripts being filtered out.

We note that filtering is a commonly used strategy to eliminate potential false positive signals in genomic data analysis. Other methods have also employed filtering. For example, Cuffdiff gives NOTEST if the number of reads is smaller than some internally defined threshold. In EBSeq, those whose 75^th^ quantile of normalized counts is less than 10 are also filtered because lowly expressed genes are more likely to be affected by noises. In our experience, transcripts with a high CV typically have low expression levels because lowly expressed transcripts generally have smaller number of reads mapped to them and this would lead to relatively higher estimation uncertainty and thus high variability of expression estimates among samples. We evaluated the impact of using various CV thresholds on filtering by examining empirical FDRs and QQ plots. Through simulations and sensitivity analysis, we chose a cutoff of 0.4, which gives satisfactory FDRs and QQ plots for all simulation settings we considered. We note that CV filtering could also help eliminate false positive results in the other methods evaluated in this paper, although these methods didn’t choose to use CV as a filter.

We note that Cuffdiff (version 2.1.1) implemented a sampling-based method for differential analysis instead of estimating the standard error of log-transformed FPKM, and this approach led to slightly better results as compared to the delta method approximation. However, the resolution of its p-value is limited by the number of samplings, and the current implementation of the program generates p-values only up to 5 × 10^−5^. When a more stringent p-value threshold is desired, the computational time will be significantly increased with an increased number of samplings.

Throughout the paper, we have used FPKMs estimated from Cufflinks as input for various evaluations. We note that meta-regression only requires estimates of isoform expression and the corresponding estimation uncertainty, but it is not tied to any particular estimation method. However, to evaluate the potential impact of isoform expression estimation method on our results, we compared the estimated isoform expression levels using three different software packages, including Cufflinks, RSEM, and MMSEQ. Our evaluation indicates that these three software packages gave highly similar expression estimates (Additional file 2). The choice of expression estimation method has little impact on our results, and this was confirmed by comparing MetaDiff with Cufflinks and MMSEQ estimated isoform expressions (Additional file 2).

In this paper, we explained the equivalence between a random-effects model that accounts for estimation uncertainty in differential expression analysis and the random-effects meta-regression. Meta-regression has been well studied in statistics and epidemiology literatures [25–27], and is easy to implement using standard software. Both RNA-Seq analysis and meta-analysis face the same problem of small sample size. The BcLR test uses a correction factor to modify the standard LR test for small sample sizes. Huizenga et al. [18] compared several testing procedures for meta-regression and showed that the BcLR test and *t*-test are the two best options. In our simulation study, we found that the BcLR test outperformed *t*-test with less conservative FDR and more power to detect DE transcripts.

Meta-regression is similar in nature to MMDIFF [16], a recently developed Bayesian random-effects model for isoform differential expression analysis. We did not include MMDIFF in our evaluations because MMDIFF can only take MMSEQ [17] expression estimates as input. However, our additional analyses indicate that results from MMDIFF and meta-regression are highly concordant. Since meta-regression is frequentist-based and yields p-values, we believe it offers a useful alternative to MMDIFF.

## Conclusion

In summary, we have proposed MetaDiff, a flexible regression framework for isoform differential expression analysis that can take into account isoform expression estimation uncertainty and variation across biological replicates, and allow for covariate adjustment. Our method can effectively control for false positives due to confounding and increase the power to detect true DE transcripts. MetaDiff can be freely downloaded from https://github.com/jiach/MetaDiff. This regression framework is flexible and can be readily extended to other study designs, e.g., paired data and time course data.

## Methods

### Notation

Let *Y*_*i*_, *i* = 1,…, *n*, denote the estimated expression level of an isoform of a gene, as represented by fragments per kilobase of transcript per million fragments sequenced (FPKM) value for subject *i*, and *σ*_0*i*_ denote the standard error of the estimated FPKM. Both *Y*_*i*_ and *σ*_0*i*_ can be obtained from programs that estimate isoform-specific gene expression (e.g., Cufflinks). The variable of interest (e.g., disease status, treatment group, etc.) is denoted by *X*_*i*_. For simplicity of presentation, we assume there is only one additional variable, *Z*_*i*_, but additional variables can be easily incorporated in the regression framework as described later in this section. When *Z*_*i*_ is correlated with both *Y*_*i*_ and *X*_*i*_, it is typically called a confounder. In differential expression analysis, the FPKM values are usually log-transformed so that their distribution is approximately normal. We approximate the standard error of log(*Y*_*i*_) using the delta method:

1 $$ Var\left[ \log \left({Y}_i\right)\right]\kern0.24em \underline {\underline{def}}\;{\sigma}_{1i}^2\approx \frac{\sigma_{0i}^2}{{\left[E\left({Y}_i\right)\right]}^2}. $$

When the FPKM values were 0, we add 0.0001 in order to avoid negative infinity in the log transformation.

### Random-effects meta-regression for testing differential expression

Isoform-specific gene expression levels cannot be directly observed in RNA-Seq, but rather have to be estimated using deconvolution-based algorithms [8–10]. A natural way to account for variable uncertainty in isoform expression estimation when testing the relationship between isoform expression and *X*_*i*_ is to use a random-effects regression model, which can be written as

$$ \log \left({Y}_i\right)={\beta}_0+{\beta}_1{X}_i+{\beta}_2{Z}_i+{u}_i+{e}_i.\kern5.3em (2) $$

Here *u*_*i*_ represents the estimation uncertainty for log(*Y*_*i*_), and *e*_*i*_ is random error. For this random-effects model, we assume: 1) *u*_*i*_ ~ *N*(0, *σ*_1*i*_^2^), 2) *e*_*i*_ ~ *N*(0, *τ* ^2^), 3) *u*_*i*_ and *e*_*i*_ are uncorrelated, i.e., Cov(*u*_*i*_, *e*_*i*_ = 0), and 4) the *n* observations are independent. We note that equation (2) is identical to the random-effects meta-regression model that is widely used in meta-analysis [16]. Because of this, we can readily carry out statistical inference using standard statistical software, such as R (http://www.r-project.org) and Stata (Stata Corp, College Station, TX). In our analysis, we used the *metatest* package in R [18].

Specifically, to test the null hypothesis of no differential expression between cases and controls, i.e., H_0_ : *β*_1_ = 0, we consider two statistical tests: *t*-test and Bartlett corrected likelihood-ratio test (BcLR). The *t*-test statistic is calculated as

$$ t={\widehat{\beta}}_1/\widehat{se}\left({\widehat{\beta}}_1\right), $$

where the estimated parameter *β*_1_ is divided by its standard error obtained from the random-effects model. Under the null hypothesis of no differential expression, the distribution of this test statistic approximately follows a *t*-distribution with *n* – 3 degrees of freedom. Because the *t*-test can have an exact sample distribution that does not depend on the asymptotic assumption, it is expected to perform well when the model assumption is correct and sample size is small, which is common in gene expression experiments using RNA-Seq.

Alternatively, we can test for differential expression using the standard likelihood ratio test. However, the corresponding test statistic may not follow a chi-squared distribution when sample size is small. To obtain more reliable results in small samples, we consider a Bartlett corrected version, the BcLR test. Let *ℓ* be the maximized log likelihood under the full model, and *ℓ*_0_ be the log likelihood under the null model. The BcLR test statistic is calculated as

$$ \mathrm{B}\mathrm{cLR} = \mathrm{B}\mathrm{C}\mathrm{F} \times 2\left(\ell \hbox{--} {\ell}_0\right), $$

where BCF is the Bartlett correction factor. Under the null hypothesis, BcLR follows a chi-squared distribution with one degree of freedom. Huizenga et al. [18] derived the BCF for meta-regression model, and demonstrated that the BcLR test outperformed the standard likelihood-ratio test.

### Data access

RNA-Seq data have been deposited in the Gene Expression Omnibus (GEO) database (accession number GSE57344).

## Additional files

Additional file 1:
**QQ plots of different tests in detecting DE isoforms.**

Additional file 2:
**Comparison of isoform expression estimates from Cufflinks, RSEM and MMSEQ.**
