# Supplementary material for: MetaDiff: differential isoform expression analysis using random-effects meta-regression
Source: BMC Bioinformatics. 2015 Jul 2;16:208. doi: 10.1186/s12859-015-0623-z (PMC4489045; doi:10.1186/s12859-015-0623-z)

**Supplementary Figure 1.** QQ plots of different tests in detecting DE isoforms. Only non-DE transcripts with CV less than 1 in both cases and controls were included for BcLR and t-test.

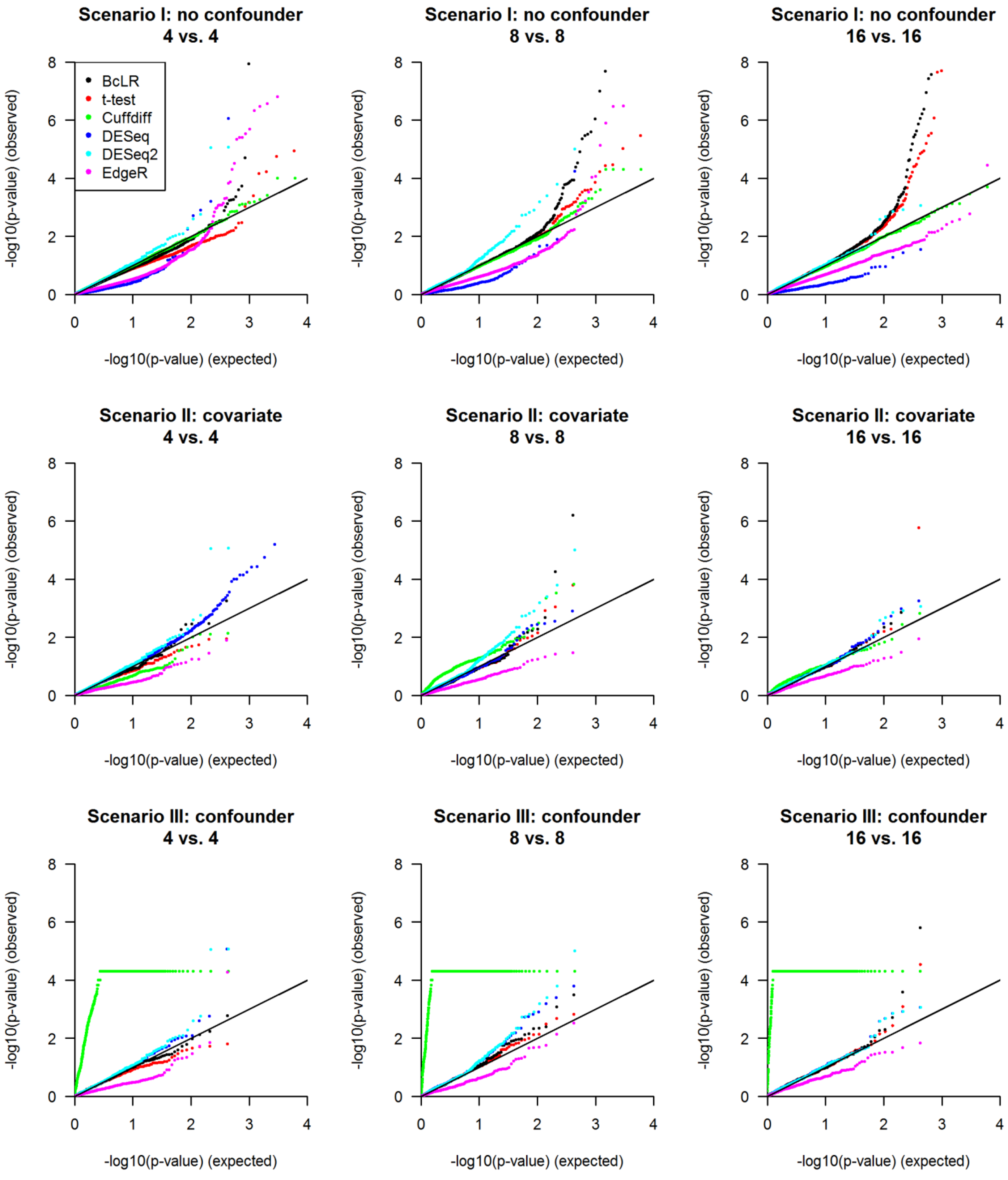

**Supplementary Figure 2.** QQ plots of different tests in detecting DE isoforms. Only non-DE transcripts with CV less than 0.9 in both cases and controls were included for BcLR and t-test.

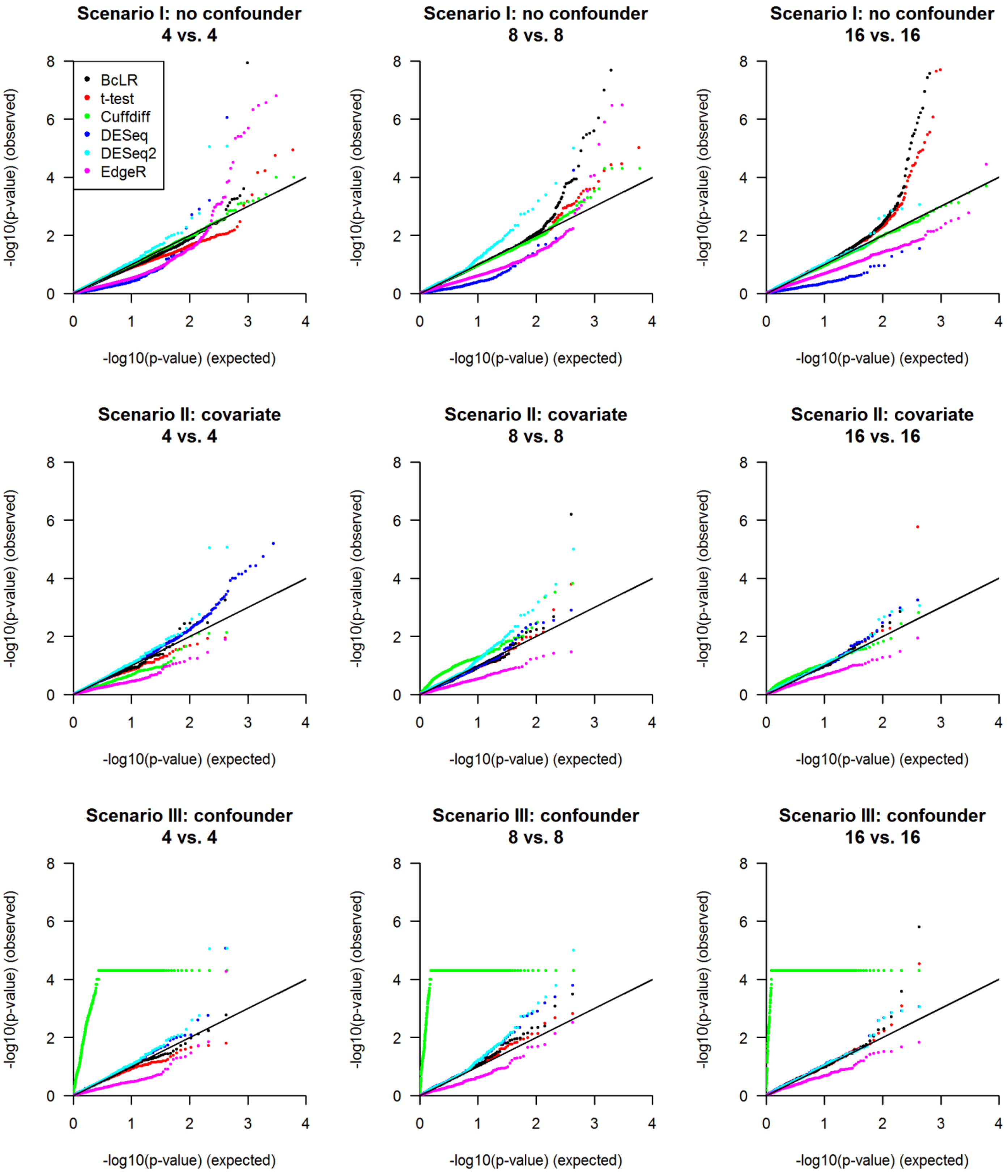

**Supplementary Figure 3.** QQ plots of different tests in detecting DE isoforms. Only non-DE transcripts with CV less than 0.8 in both cases and controls were included for BcLR and t-test.

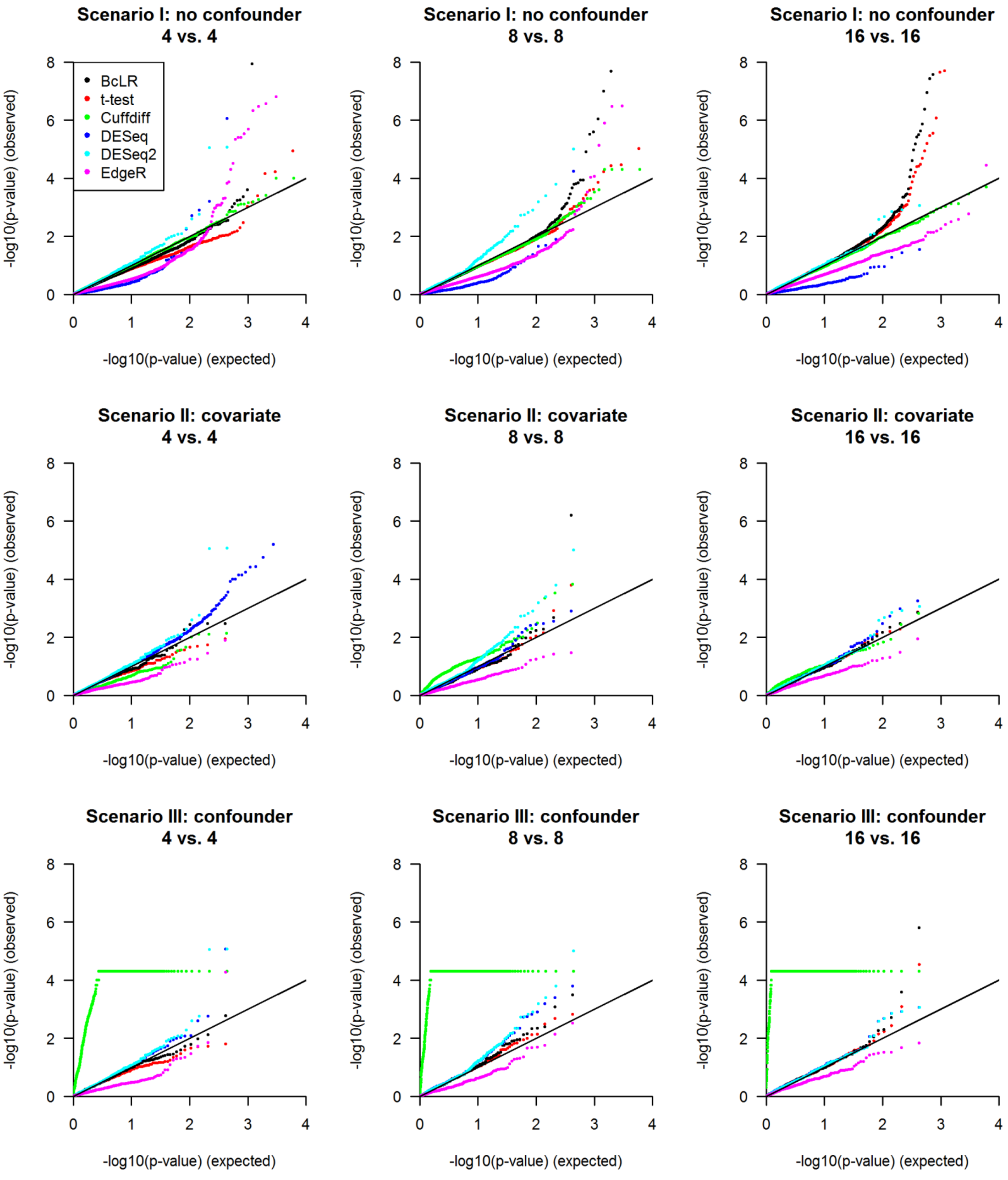

**Supplementary Figure 4.** QQ plots of different tests in detecting DE isoforms. Only non-DE transcripts with CV less than 0.7 in both cases and controls were included for BcLR and t-test.

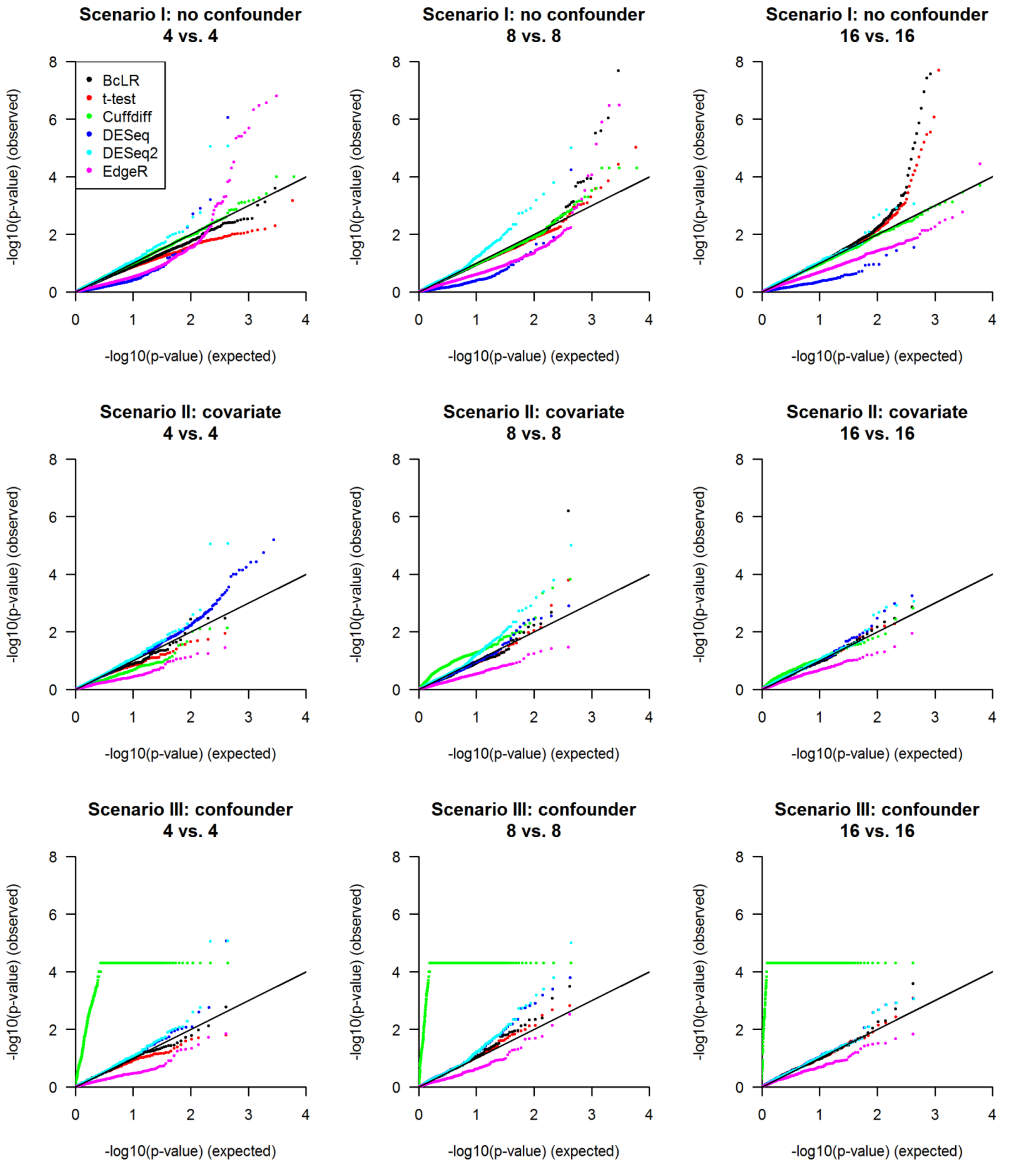

**Supplementary Figure 5.** QQ plots of different tests in detecting DE isoforms. Only non-DE transcripts with CV less than 0.6 in both cases and controls were included for BcLR and t-test.

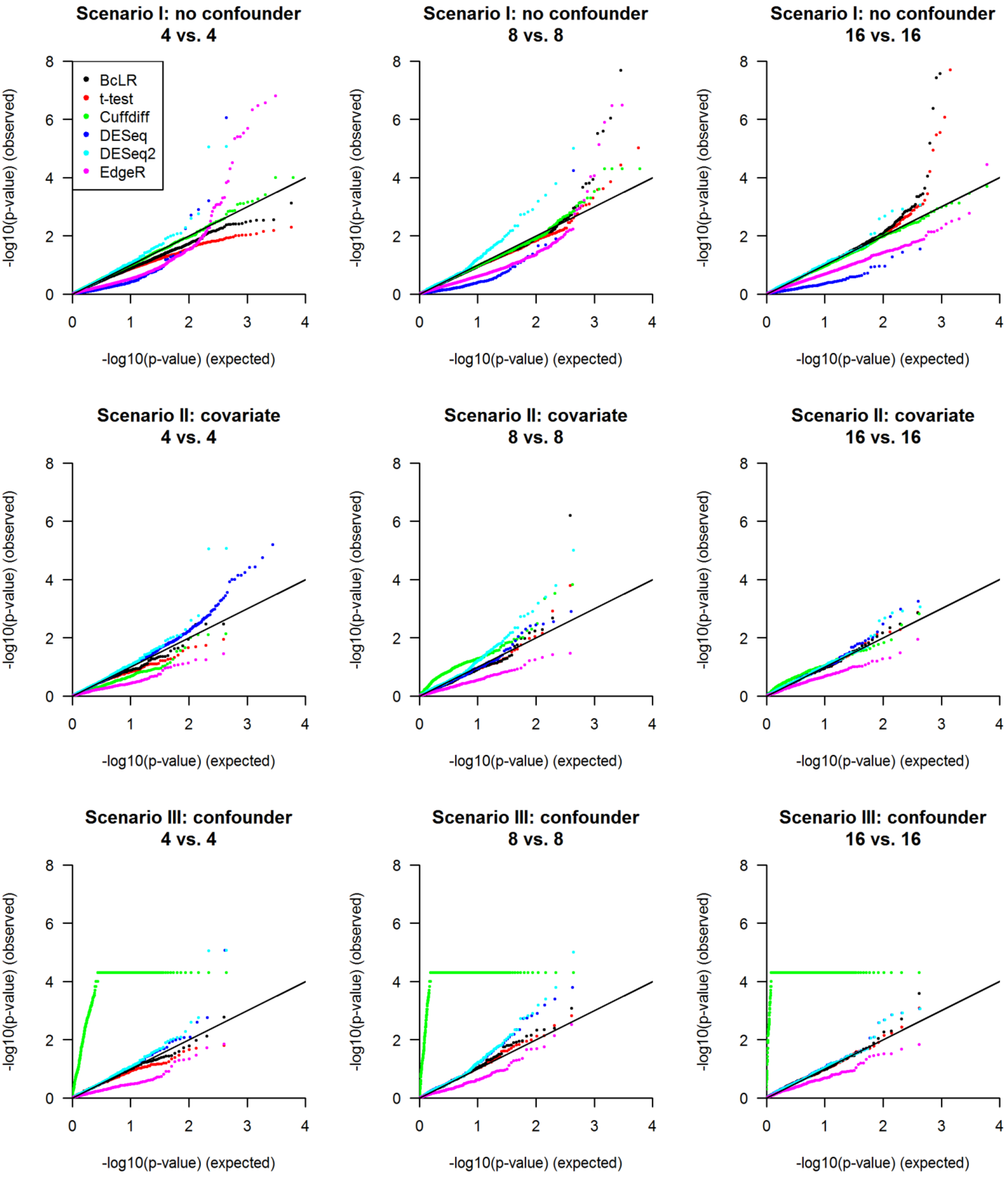

**Supplementary Figure 6.** QQ plots of different tests in detecting DE isoforms. Only non-DE transcripts with CV less than 0.5 in both cases and controls were included for BcLR and t-test.

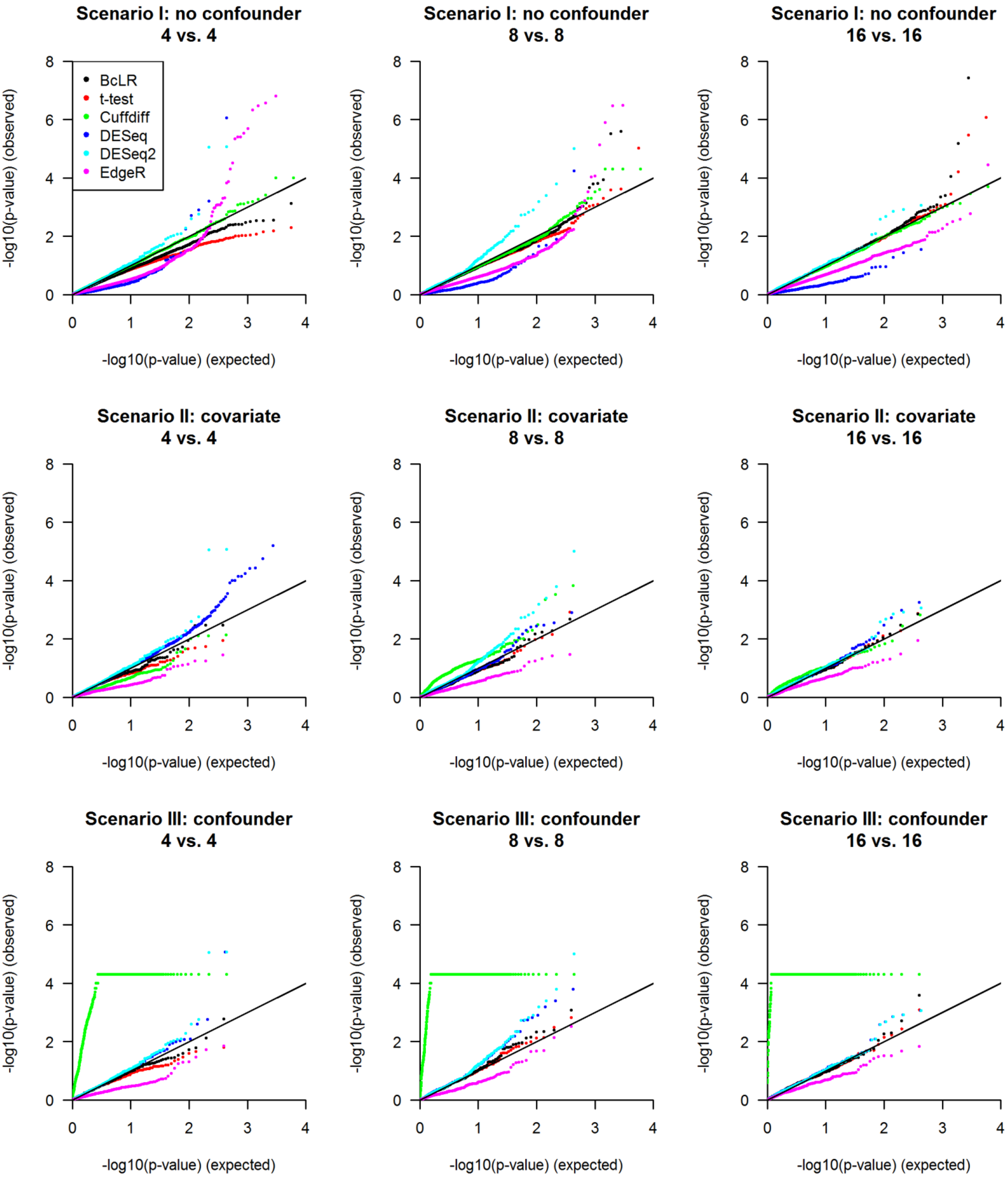

**Supplementary Figure 7.** Estimated FDRs for the BcLR test at various nominal FDR levels (0.01 to 0.1 with 0.01 increment). Only transcripts with CV less than given cutoff in both cases and controls were included.

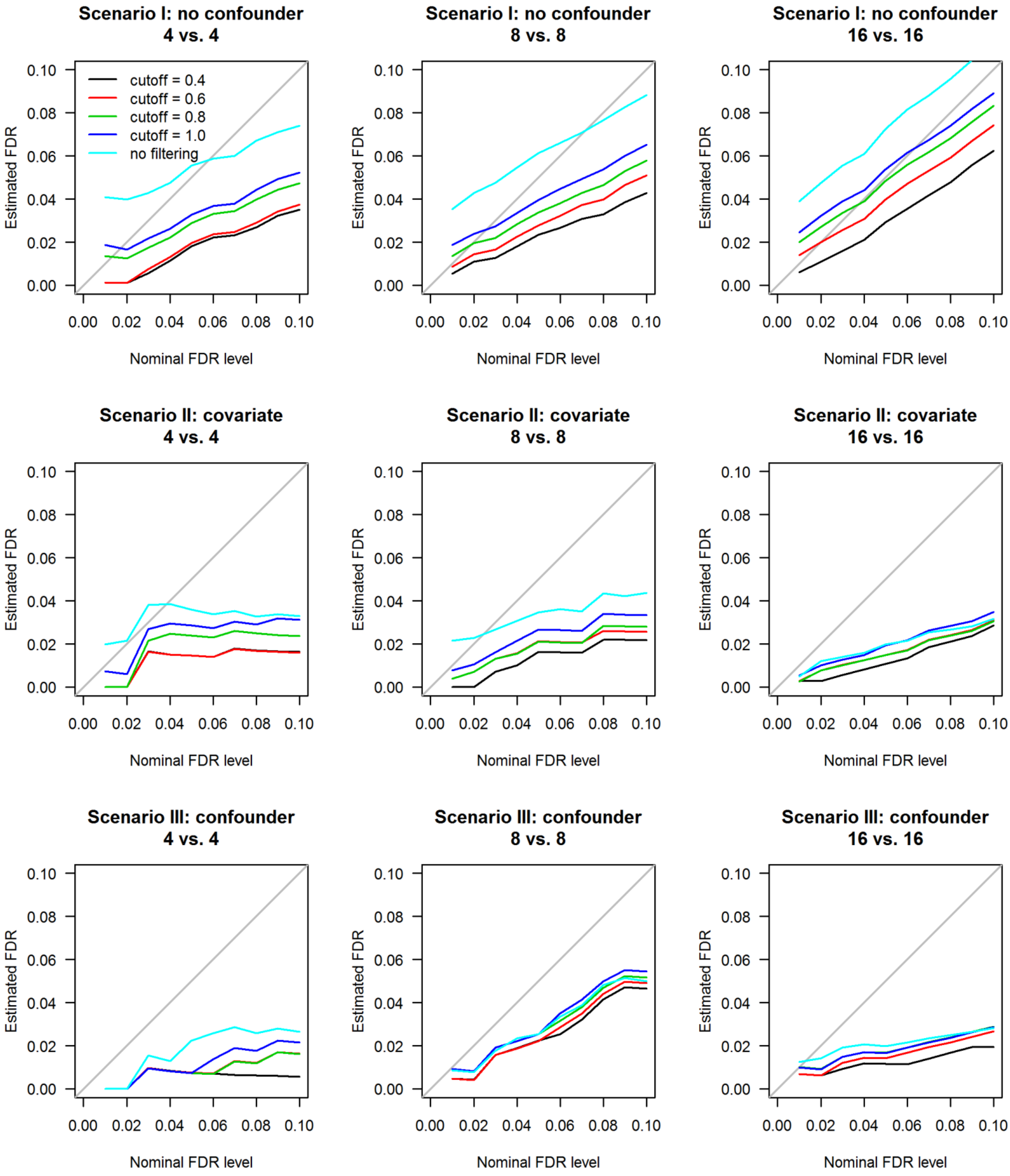

**Supplementary Figure 8.** Receiver Operating Characteristic (ROC) curves. Sensitivity and specificity were calculated by varying the p-value cutoffs.

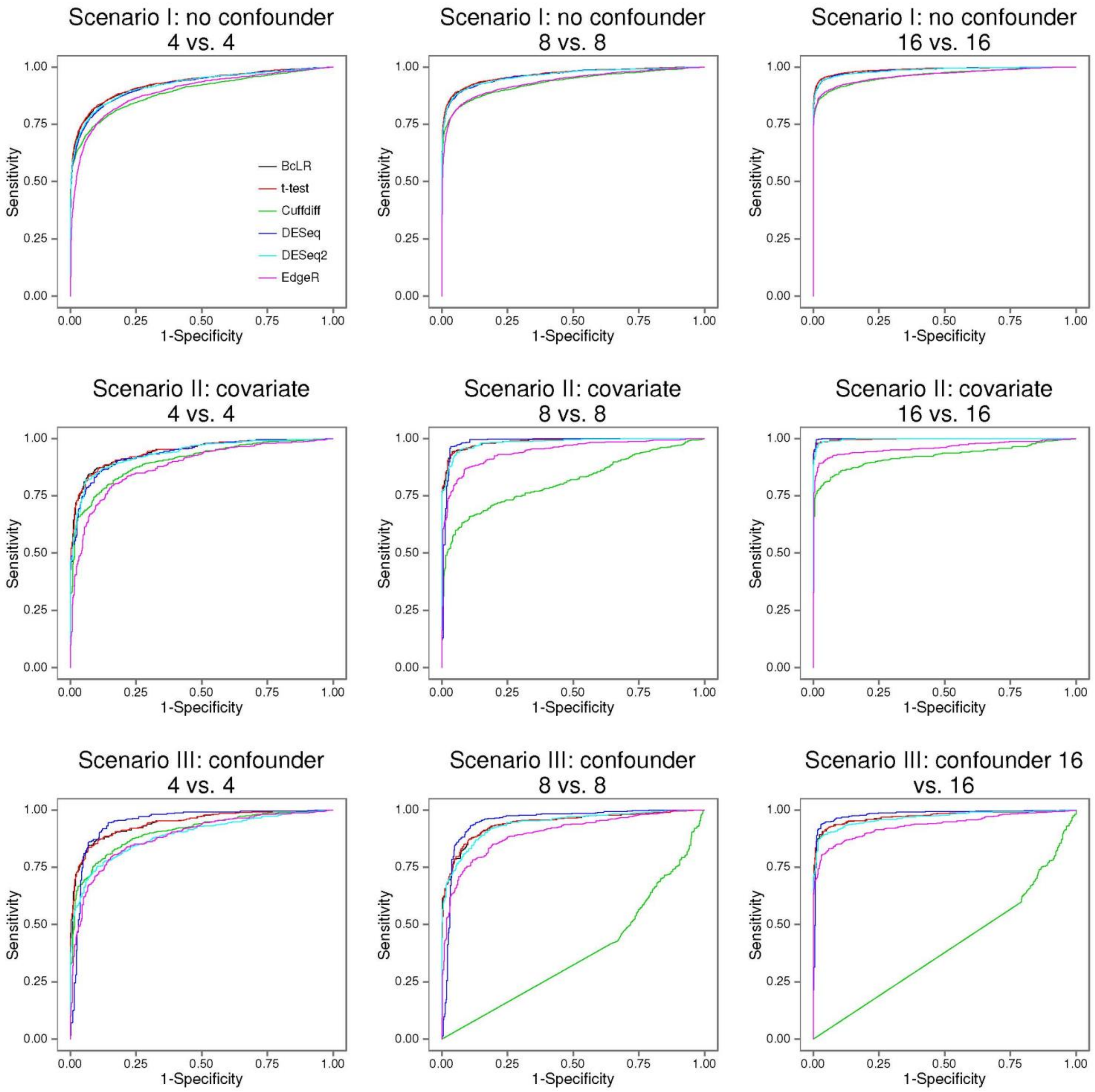

Supplement: Additional file 1: — QQ plots of different tests in detecting DE isoforms. [file 12859_2015_623_MOESM1_ESM.pdf]
