# Supplementary material for: MetaDiff: differential isoform expression analysis using random-effects meta-regression
Source: BMC Bioinformatics. 2015 Jul 2;16:208. doi: 10.1186/s12859-015-0623-z (PMC4489045; doi:10.1186/s12859-015-0623-z)
Supplement: Additional file 2: — Comparison of isoform expression estimates from Cufflinks, RSEM and MMSEQ. [file 12859_2015_623_MOESM2_ESM.pdf]

Supplementary Text

Comparison of isoform expression estimates from Cufflinks, RSEM and MMSEQ

We simulated ~10 million 76 bp paired-end reads from the Flux Simulator. Isoform expression was estimated using Cufflinks, RSEM and MMSEQ, respectively. Since RSEM has compatibility issues with the RefSeq gene annotations, we simulated a new sample based on Ensembl genes and isoforms. We calculated Pearson correlation coefficient of the estimated isoform expression levels (on log10(FPKM) scale) between two different methods. The correlation coefficient was calculated based on isoforms that have FPKM >1 in all three methods. We note that MMSEQ does not explicitly estimate FPKMs, but as suggested by the authors, exp(log_mu) can be considered as an isoform expression level estimate. So in our calculations, we used exp(log_mu) for MMSEQ.

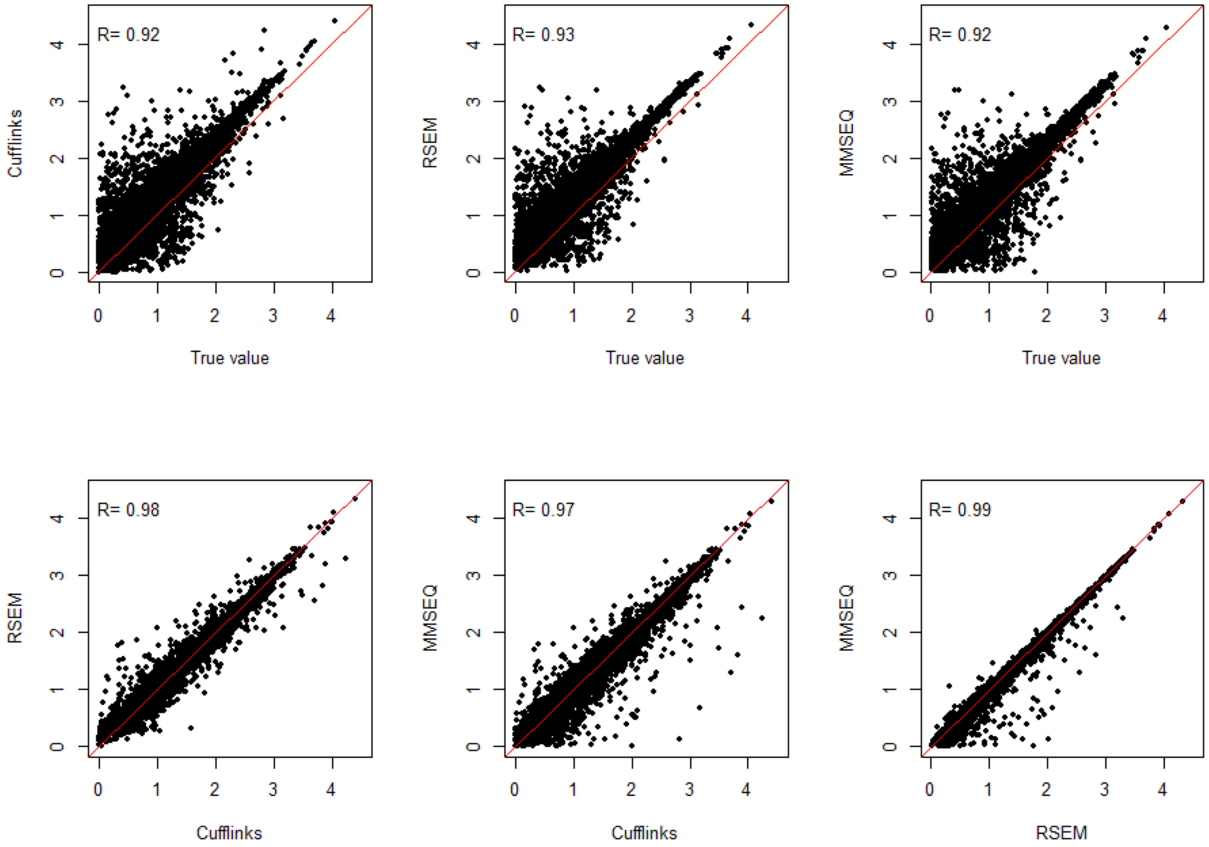

Since a large portion of the isoforms are not expressed or expressed at very low levels, we also compared the proportions of isoforms with isoform expression FPKM<1. The proportions are 0.26 (true values), 0.24 (Cufflinks), 0.23 (RSEM), and 0.29 (MMSEQ), respectively.

We also applied different FPKM cutoff values in our calculations and obtained similar results.

FPKM > 0.1:

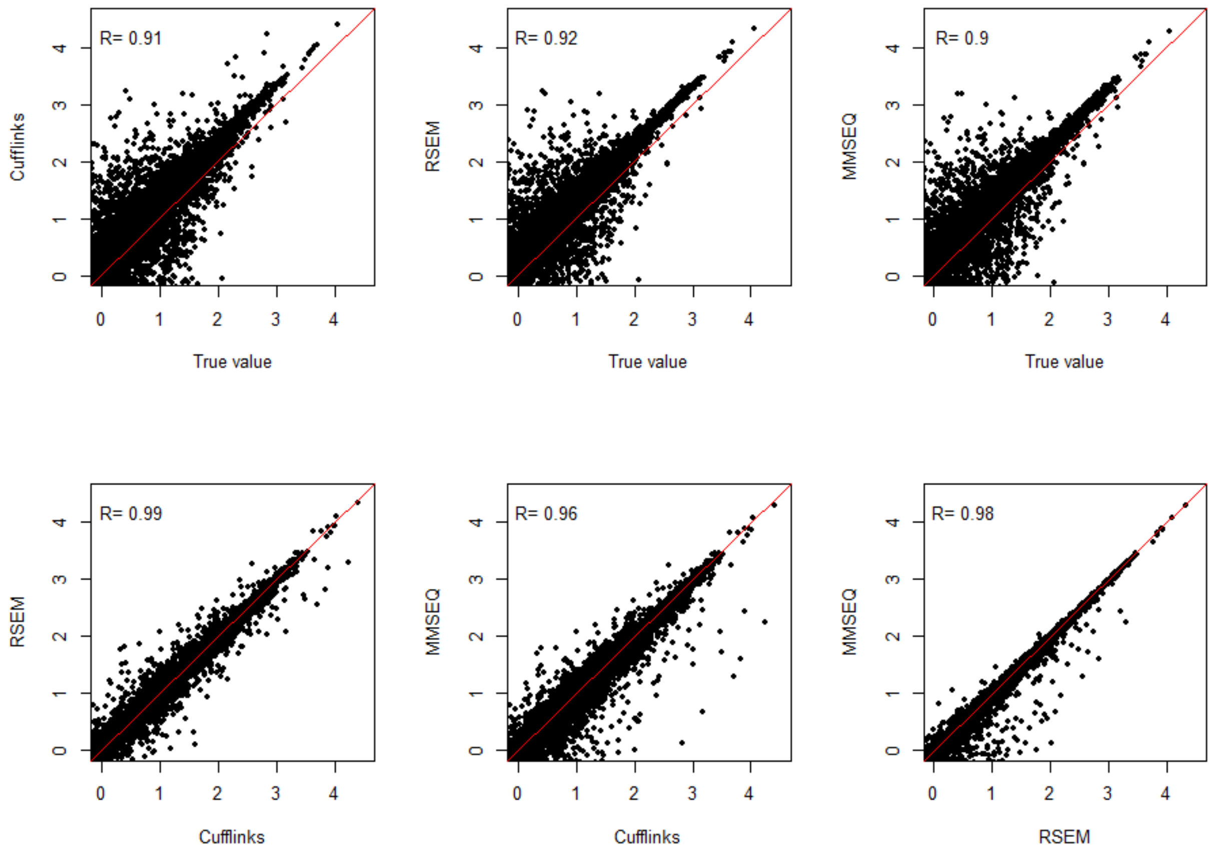

The proportions of isoforms with isoform expression FPKM<0.1 are 0.03 (true values), 0.13 (Cufflinks), 0.13 (RSEM), and 0.18 (MMSEQ), respectively.

Overall, our results indicate that Cufflinks, RSEM and MMSEQ produce highly similar FPKM estimates, and thus we expect to get similar results with RSEM or MMSEQ FPKM values as input.

Comparison of MetaDiff results with MMSEQ and Cufflinks estimated FPKMs

We analyzed the simulated data using MMSEQ's estimated isoform expression as input for MetaDiff, and compared the results with input obtained from Cufflinks estimates. In the figure below, we generated scatter plot of –log10(p-value). As expected, the results obtained from MMSEQ and Cufflinks are highly concordant. The correlation of the –log10 transformed p-values is 0.97.

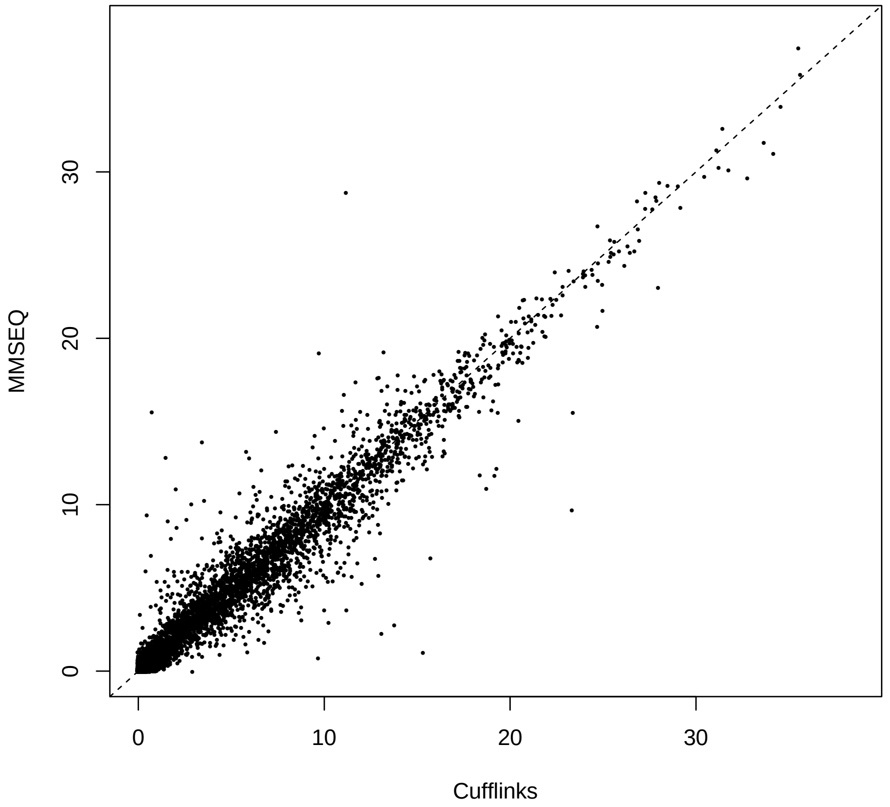

Comparison between MMDIFF and meta-regression

Since MMDIFF is also based on random-effects model but uses a Bayesian approach, we expect its performance to be similar to meta-regression. To confirm this, we considered samples simulated under Scenario I. We used MMSEQ estimated isoform expression and the corresponding estimation uncertainty as input for meta-regression. Since MMDIFF does not give p-values, one way to assess the degree of concordance with meta-regression is to calculate the Spearman correlation coefficients between MMDIFF's posterior probability of non-DE and meta-regression p-values. The correlation coefficient between MMDIFF and BcLR test is 0.77, and the corresponding correlation coefficient between MMDIFF and t-test is 0.78. The relatively high Spearman correlation coefficients suggest that the results between MMDIFF and meta-regression are concordant. For isoforms that were found to be DE by both MMDIFF (posterior probability of DE>0.95) and meta-regression (FDR adjusted p-value<0.05), we compared the distributions of the meta-regression p-values and MMDIFF's posterior probabilities. As shown below, the distributions are highly similar.

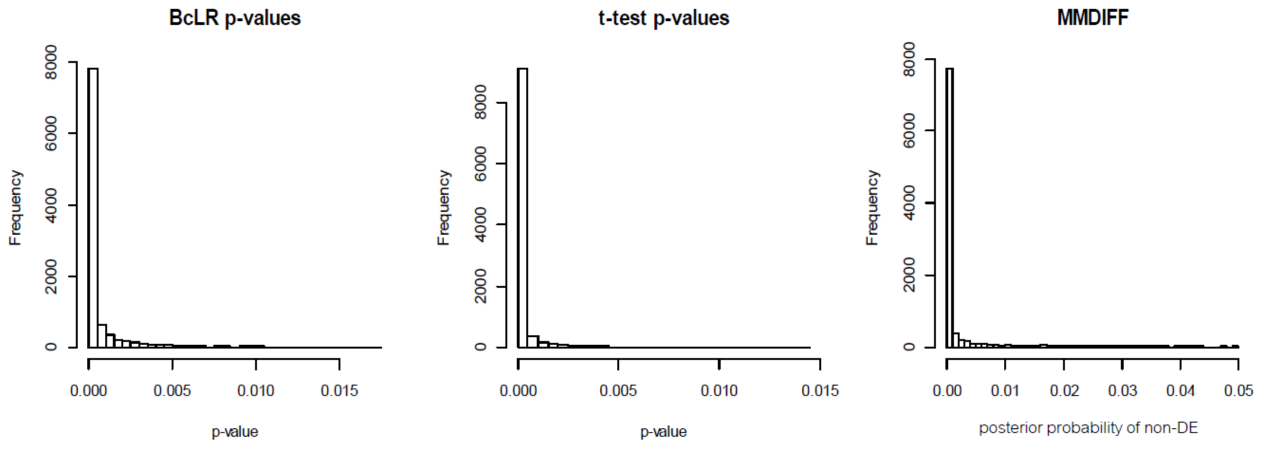

Since MMDIFF can only take MMSEQ estimates as input, we chose not to include it in our comparison with other methods because other programs such as Cuffdiff requires BAM files as input. However, given the similarity between MMDIFF and meta-regression, we believe that one would draw a similar conclusion if MMDIFF was used in the analysis.

Supplementary Table 1 - Percentage of true DE transcripts (at 0.05 FDR level) that were filtered out by CV for BcLR and t-test. We required CV to be less than 0.4 in both cases and controls.

	**Scenario I: no confounding**				**Scenario II: age as a covariate**				**Scenario III: age as a confounder**		
	4 vs. 4	8 vs. 8	16 vs. 16		4 vs. 4	8 vs. 8	16 vs. 16		4 vs. 4	8 vs. 8	16 vs. 16
BcLR	1.33	2.77	3.72		1.70	7.59	9.81		1.06	2.27	4.85
t-test	0.65	2.69	3.69		0.64	7.16	10.02		0.00	2.06	4.64
